# Transcatheter treatment by valve-in-valve and valve-in-ring implantation for prosthetic tricuspid valve dysfunction

**DOI:** 10.1007/s00508-021-01842-x

**Published:** 2021-03-31

**Authors:** Varius Dannenberg, Carolina Donà, Matthias Koschutnik, Max-Paul Winter, Christian Nitsche, Andreas A. Kammerlander, Philipp E. Bartko, Christian Hengstenberg, Julia Mascherbauer, Georg Goliasch

**Affiliations:** Department of Cardiology, Medical University of Vienna, Waehringer Guertel 18–20, 1090 Vienna, Austria

**Keywords:** Valve degeneration, Surgical valve repair/replacement, Deterioration of annuplasty/prosthesis, Minimale invasive procedures, Tricuspid regurgitation/stenosis

## Abstract

Valve degeneration after surgical tricuspid valve replacement or repair is frequent and may require repeat replacement/repair. For high-risk patients, transcatheter valve-in-valve and valve-in-ring procedures have emerged as valuable treatment alternatives. Preprocedural transthoracic echocardiography is the method of choice to detect malfunction of the prosthesis including degenerative stenosis and/or regurgitation requiring reintervention. Subsequently, computed tomography is helpful for detailed anatomical analysis and periprocedural planning. Device selection and sizing depend on the size and structural details of the implanted ring or prosthesis. The procedure is mainly guided by fluoroscopy; however, transesophageal echocardiography provides complementary guidance during device implantation. Preferred access route is the right femoral vein but in cases of more horizontal implants a jugular approach might be feasible. Suitable transcatheter valves are the Edwards Sapien 3 and the Medtronic Melody valves. Differences in surgical prostheses or annuloplasty implants are important for device selection, height consideration and additional ballooning prior to or after implantation. Transesophageal echocardiography postimplantation is convenient for the assessment of transvalvular gradients or paravalvular leaks.

**Supplementary Information:**

The online version of this article (10.1007/s00508-021-01842-x) contains supplementary material, which is available to authorized users.

## Introduction

Valve degeneration after tricuspid valve repair or replacement is frequent and can require tricuspid valve r‑intervention [1]; however, recent data illustrate that isolated tricuspid valve interventions inhere a significant procedural risk with in-hospital mortality rates ranging around 10% and major complications affecting up to one third of all patients [2]. Consequently, tricuspid resurgery is rarely performed and transcatheter tricuspid valve-in-ring (TViR) and valve-in-valve (TViV) repair have evolved as promising treatment options for the majority of patients who would otherwise remain untreated [3, 4]. These advances have been further supported by the recent American College of Cardiology (ACC)/American Heart Association (AHA) guidelines update for the management of patients with valvular heart disease that endorse catheter-based treatment for prosthetic valve dysfunction in high-risk patients with both bioprosthetic stenosis and/or regurgitation and feature this recommendation among their top 10 take-home messages [5]. Principles, techniques and considerations of TViR and TViV are discussed in this review.

## Preprocedural assessment

Meticulous preprocedural planning is key for a successful TViR/TViV intervention. Transthoracic echocardiography (TTE) is the primary imaging modality for the diagnostics of degenerated tricuspid protheses and failed ring repairs. Recent guidelines define clinically significant tricuspid stenosis as a mean transvalvular gradient of ≥ 5 mm Hg [6]; however, in tricuspid bioprostheses the mean gradient often exceeds 5 mm Hg, wherefore a mean transvalvular gradient of ≥ 10 mm Hg might be more suitable to define significant stenosis [7].

Nevertheless, it is important to note that transvalvular gradients are heart rate dependent and may also be elevated in the setting of a patient-prosthesis mismatch. Therefore, comparison with previous examinations is of particular significance to correctly differentiate degenerative prosthesis stenosis from patient-prosthesis mismatch or valve thrombosis. Furthermore, transesophageal echocardiography (TOE) can be particularly useful to assess leaflet motion or, in cases with good image quality, estimation of valve opening area by planimetry may be possible.

Assessment of right ventricular size and function is of particular significance in the planning process prior to TViR/TViV interventions. TTE is the first-line imaging modality for RV assessment using an integrative approach and multiple windows. A comprehensive echo assessment of right ventricular function (RVF) may encompass beside a thorough visual assessment, the measurement of tricuspid annulus plane systolic excursion (TAPSE), tissue Doppler systolic tricuspid annular velocity (TDI S’), RV free-wall longitudinal strain and right ventricular fractional area change (RVFAC). In a recent analysis of patients undergoing tricuspid valve repair, a reduced RVF measured by TAPSE, was associated with worse outcome [8]; however, following tricuspid annuloplasty TAPSE may be inaccurate because of reduced annulus motion and should be used with caution for functional assessment prior to TViR interventions [9]. Cardiac magnetic resonance (CMR) imaging might be a valuable complement for assessment of RV function particularly in patients where the imaging quality is limited. A recent analysis of patients undergoing isolated tricuspid valve surgery confirmed right heart failure signs and right ventricular dysfunction as significant independent predictors of all-cause mortality [2]; however, future studies need to identify which patients might benefit from TViR/TViV interventions and where these procedures might be futile.

**Fig. 1:**
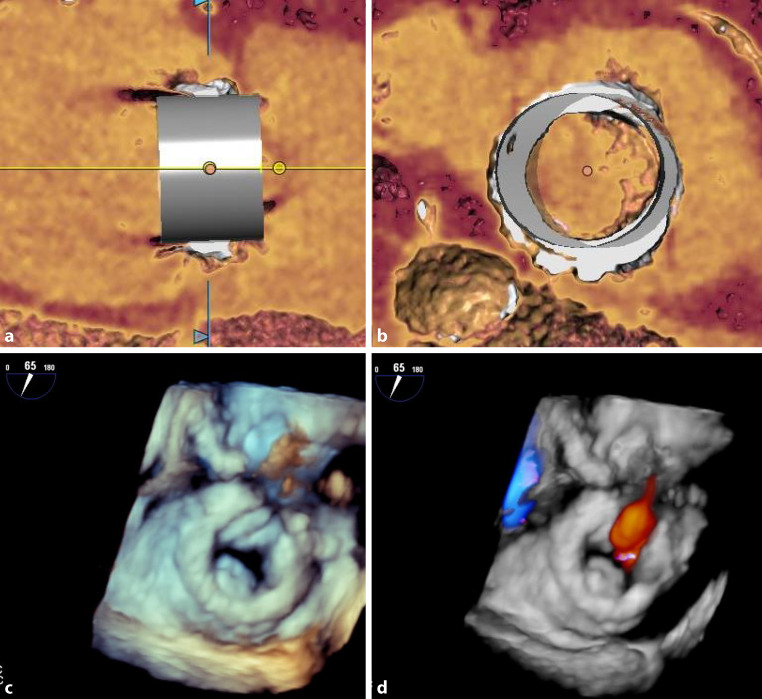
Simulation of an implanted transcatheter heart valve in a long axis (**a**) and short axis (**b**) using CT and specific software (3mensio Structural Heart, Pie Medical Imaging, Maastricht, The Netherlands). Preprocedural early diastolic image of the degenerated bioprosthesis from the atrium using 3D transesophageal echocardiography (**c**, supplemental video 1) and midsystolic color Doppler, showing tricuspid regurgitation (**d**, supplemental video 2)

A reoperation is also indicated in cases of severe tricuspid regurgitation, evaluated by echocardiography according to recent guidelines [10]. Further procedural planning includes correct sizing of the valve according to recommendations for the specific bioprosthesis or the annuloplasty ring, as provided by the manufacturer. Computed tomography (CT) can add detailed anatomical information and a three-dimensional reconstruction using a specific software can simulate the implantation of the transcatheter heart valve (3mensio Structural Heart [Pie Medical Imaging, Maastricht, The Netherlands], Fig. 1a, b). If pulmonary hypertension is suspected, a right heart catheterization should be performed prior to the procedure. Patients with tricuspid regurgitation and severe pulmonary hypertension are at high risk for right heart failure after tricuspid valve repair [5].

## Step-by-step guide for tricuspid valve-in-valve and valve-in-ring procedures

Vascular access is gained via the jugular or femoral vein. In the case of a vertical tricuspid valve orientation the femoral access route should be preferred, and in the case of a more horizontal valve orientation, the jugular vein is a more suitable choice [11]; however, novel delivery systems are more versatile, allowing implantation of a valve via the femoral vein despite an obliquely positioned tricuspid valve. Alternatively, a transatrial approach for TViV might be used but this access route is more complex, including minithoracotomy and double lumen intubation. Therefore, this approach should be used only in very specific conditions, such as unfavorable venous anatomy or a combination of inferior vena cava thrombosis and a vertical or oblique bioprosthesis or ring, which cannot be reached by a jugular access route [12, 13].

The procedure may be performed with the patient under conscious sedation and local anesthesia. Still, this excludes the usage of transesophageal echocardiography (TEE), which is helpful for intraprocedural reassessment of the degenerated valve (Fig. 1c, d, supplemental videos 1, 2) and positioning of the wires and the prosthesis.

Crossing of the tricuspid valve might be challenging, especially in the case of a very small valve orifice or a severely dilated right atrium. Adjusting fluoroscopy in an angle showing an en-face or end-on view of the prosthesis is in most cases convenient for guiding of the catheter. A multipurpose catheter is used in combination with a shaped wire to guide the catheter tip towards the tricuspid valve. A steerable sheath (e.g. Agilis NXT, Abbott Vascular–Structural Heart, Menlo Park, CA, USA) in combination with a 0.035 inch exchange-length stiff-angled Glidewire (Terumo Interventional Systems, Somerset, NJ, USA) can be useful if crossing the prosthesis is challenging and enables a secure catheter position to be maintained during deployment of the guidewire into the apex of the right ventricle.

After crossing of the prosthesis/ring, two different wire positions may be used: (i) pulmonary artery (PA) wire position or (ii) RV loop:

i. To achieve a PA wire position the distal PA branch is wired with a Glidewire and a catheter (e.g. multipurpose) located deep in the vessel. The left PA is preferred as it provides a more coaxial rail for delivery of the transcatheter heart valve. Once stable position of the catheter is achieved, the Glidewire is exchanged for an exchange-length 0.035-inch Amplatz Super Stiff guidewire (Boston Scientific, Marlborough, MA, USA) or a 0.035-inch double-curved Lunderquist (Cook Medical, Bloomington, IN, USA). Specific attention should be paid to the tips of the stiff guidewires to prevent damage of the PA.
ii. The RV apical wire technique is facilitated by a steerable sheath to maintain catheter position (e.g. pig-tail catheter) in the RV apex during advancement of a stiff wire with a preformed ventricular loop such as the Safari wire (Boston Scientific). Although using a RV wire loop may be preferable to allow better coaxiality of the THV, this technique typically requires a dilated right ventricle to place the wire loop.

In general, a surgical prosthesis with a diameter of ≤ 25 mm is suitable for a Melody valve (Medtronic, Minneapolis, MN, USA) and a diameter of ≥ 29 mm for a Sapien 3 (Edwards Lifesciences, Irvine, CA, USA). Currently available online applications can be used for identification of a suitable device (App Store/Google Play Store: ViV Mitral, UBQO Limited, London, UK). The Sapien 3 has longer leaflets and a taller stent height, which allows overexpansion up to a diameter of 31 mm [14]. Furthermore, it is crucial that the valve is mounted for an antegrade delivery onto the delivery catheter and introduced into the sheath with the Edwards E logo facing downward to allow a correct flexion of the catheter. The Melody valve is mounted in a regular way as for pulmonary valve implantation. For balloon sizing of the existing prosthesis, a 22 mm balloon is typically used to figure the waist of the valve prior to implantation.

**Fig. 2:**
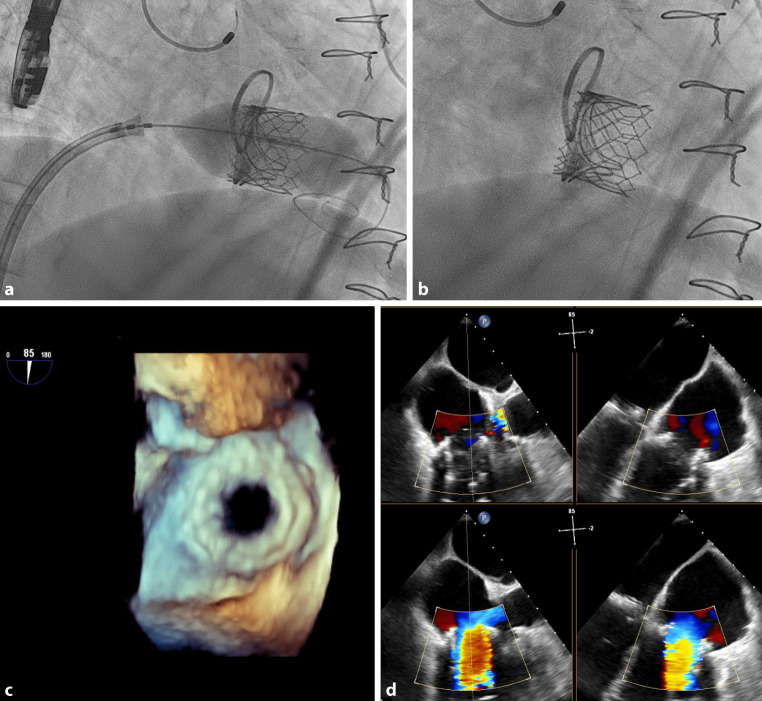
Fluoroscopy of the balloon implantation (**a**, supplemental video 3) and the final result (**b**, supplemental video 4), postprocedural early diastolic image using echocardiography, the stent of the transcatheter valve is visible (**c**, supplemental video 5), postprocedural biplane imaging midsystolic (**d**, *upper images*, supplemental video 6) and early diastolic (**d**, *lower images*, Supplemental video 6)

If the crossing of the prosthesis is difficult, the Sapien 3 delivery catheter pusher can be retracted, to make the distal part more flexible, or the balloon can be slightly inflated for better tracking of the system. In some rare cases of very stenotic valves, predilation of the prosthesis might be considered. In cases of TViV and a prosthesis with visible stent frame, the central marker of the Sapien 3 should be positioned 3–5 mm below the base of the surgical valve stent frame in the ventricle (Fig. 2a, b, supplemental videos 3, 4). If a prosthesis displays outflow markers only, the central marker is positioned 2 mm ventricularly. In cases of no radiopaque markers, the base of the central marker should be positioned at the annulus plane. In TViR, the central marker of the Sapien 3 should be adjusted 2 mm ventricular to the ring. The Melody valve is implanted with dilatation of the inner balloon and observing a height at which 40% of the stent frame is in the right atrium. The inflation of the outer balloon aligns the valve into the correct tricuspid position. After implantation, meticulous evaluation of the newly implanted valve can be performed using TEE (Fig. 2c, d, supplemental videos 5, 6).

For TViR a few considerations are important: 1) is a ring or a band implanted, 2) is the implant flexible, semirigid or rigid and 3) is it complete or incomplete. Incomplete and semirigid or flexible implants are generally more challenging for proper anchoring of the THV. Balloon sizing prior to implantation is of major importance. On the one hand, flexible implants tend to require more overexpansion and, on the other hand, semirigid and rigid implants are rather prone to perivalvular leakage. Incomplete rings or bands tend to have a higher risk of valve embolization, which can be avoided by a cautious and slow positioning of the THV.

## Specific considerations

Jailing of pacemaker leads is possible but should be done with caution, especially in patients who are pacemaker dependent [15, 16]. Other options, such as epicardial leads or left ventricular pacing should be considered prior to TViR or TViV. If jailing is the preferred option, a functional test is recommended immediately after implantation of the THV.

In the case of excessive cardiac motion, valve stabilization during deployment is inevitable. This can be achieved by rapid pacing of the ventricle. Implanted pacemaker leads can be used for this maneuver. If not available, right atrial or coronary sinus pacing via a temporary pacemaker lead is the method of choice.

In some specific cases, surgeons may be confronted with paravalvular leakage of the surgical prosthesis or ring and an insufficient functioning valve. In this case, a stable implantation of the THV prior to perivalvular leakage closure by an appropriate device is recommended.

## Illustrative example of the first TViV case in Austria

A 66-year-old male patient presented with dyspnea NYHA functional class III, dizziness and syncope. Due to an autoimmune nephritis, the patient was on peritoneal dialysis and was evaluated for kidney transplantation. Leg edema was present on physical examination. The medical history included the implantation of a dual-chamber epicardial pacemaker because of a complete atrioventricular (AV) block 21 years ago, and mitral valve reconstruction with annuloplasty (Physio II 28 mm, Edwards Lifescience) 3 years ago, as well as tricuspid valve replacement (Perimount 29 mm, Edwards Lifescience) 5 years ago.

Coronary angiography was unremarkable, and the pacemaker showed proper settings and function. Transthoracic echocardiography showed a moderately reduced biventricular function. The reconstructed mitral valve showed mild regurgitation and a mean transvalvular gradient of 5 mm Hg. The tricuspid valve bioprosthesis was degenerated with reduced leaflet motion, a mean transvalvular gradient of 11 mm Hg, a pressure half-time of 340 ms, and moderate tricuspid regurgitation (Fig. 1c, d, supplemental videos 1, 2). In contrast, 1 year after surgery the mean transvalvular gradient was 5 mm Hg and the pressure half-time was 134 ms. Planimetry of valve opening area was not possible because of poor image quality and shadowing of the bioprosthesis.

The aortic valve showed no pathology and no significant pulmonary hypertension was detected. All baseline characteristics are displayed in Table 1**.**

**Table 1:** Clinical characteristics of the first patient undergoing tricuspid valve-in-valve intervention in Austria

	Baseline	
**Clinical parameters**		
Age (years)	66	–
Sex	Male	–
BMI	36	–
NYHA functional class	III	–
**Laboratory values**		
NT-proBNP (pg/ml)	1898	–
Creatinine (mg/dl)	11.4	–
**Echocardiography**		
LVEDV (ml)	103	–
LVEF (%)	41	–
RVEDD (mm)	38	–
RA volume (ml)	112	–
TAPSE (mm)	15	–
FAC (%)	33	–
sPAP (mm Hg)	29	–
**Procedural echocardiography**	Preimplantation	Postimplantation
Mean transvalvular gradient (mm Hg)	11	4
Pressure half-time (ms)	340	150

*NYHA* New York Heart Association, *NT-proBNP N*-terminal prohormone of brain natriuretic peptide, *LVEDV* left ventricular end-diastolic volume,* LVEF* left ventricular ejection fraction, *RVEDD* right ventricular end-diastolic diameter,* RA* right atrium,* TAPSE* tricuspid annular plane systolic excursion,* FAC* fractional area change,* sPAP* systolic pulmonary artery pressure, *BMI* body mass index

The interdisciplinary heart-team of our center favored an interventional approach due to a EuroScore II of 11.6% and chronic renal failure with peritoneal dialysis. An Edwards Sapien 3, 29 mm valve was deemed suitable for valve-in-valve implantation into the Edwards Perimount 29 mm prosthesis. The implantation was guided by TEE and fluoroscopy (Fig. 2a, b, supplemental videos 3, 4). The mean transvalvular gradient after the successful intervention was 4 mm Hg and the pressure half-time was 150 ms, without tricuspid regurgitation (Fig. 2c, d, supplemental videos 5, 6). The postinterventional course of the patient was prolonged by a hospital-acquired pneumonia. The discharge echocardiogram showed a well-functioning valve with no valvular/paravalvular regurgitation and the patient could be discharged in an improved clinical condition.

## Conclusion

The TViV and TViR are safe alternatives to surgical redo operations. A TTE is the key for identifying malfunctioning valves and CT is convenient for correct valve sizing. There is a certain variation of surgical implants, and therefore a correct selection of wires, balloons and access route is important. Generally, appropriate valves are the Sapien 3 and the Melody valves depending on the patient’s specific anatomical situation.

## Supplementary Information

**Supplemental video 1: **Preprocedural 3D transesophageal echocardiography of the tricuspid valve from the atrial side, showing a degenerated bioprosthesis with restricted opening.

**Supplemental video 2: **Preprocedural color Doppler 3D transesophageal echocardiography of the bioprosthesis with a significant central regurgitant jet.

**Supplemental video 3: **Fluoroscopic movie displaying the deployment of the transcatheter valve in the degenerated bioprosthesis.

**Supplemental video 4: **Final fluoroscopic result of the deployed transcatheter valve.

**Supplemental video 5: **Identical image acquisition as displayed in supplemental video 1 at the end of the procedure displaying the deployed transcatheter valve.

**Supplemental video 6:** Postprocedural color Doppler biplane acquisition, showing a well-functioning valve without valvular or paravalvular regurgitation.
